# Gentamicin Sulfate PEG-PLGA/PLGA-H Nanoparticles: Screening Design and Antimicrobial Effect Evaluation toward Clinic Bacterial Isolates

**DOI:** 10.3390/nano8010037

**Published:** 2018-01-12

**Authors:** Rossella Dorati, Antonella DeTrizio, Melissa Spalla, Roberta Migliavacca, Laura Pagani, Silvia Pisani, Enrica Chiesa, Bice Conti, Tiziana Modena, Ida Genta

**Affiliations:** 1 Department of Drug Sciences, University of Pavia, 27100 Pavia, Italy; rossella.dorati@unipv.it (R.D.); antonella.detrizio01@universitadipavia.it (A.D.); silvia.pisani01@universitadipavia.it (S.P.); enrica.chiesa01@gmail.com (E.C.); tiziana.modena@unipv.it (T.M.); Ida.genta@unipv.it (I.G.); 2 Department of Clinical-Surgical, Diagnostic and Pediatric Sciences, Unit of Microbiology and Clinical Microbiology, University of Pavia, 27100 Pavia, Italy; melissa.spalla@unipv.it (M.S.); roberta.migliavacca@unipv.it (R.M.); laura.pagani@unipv.it (L.P.)

**Keywords:** nanoparticles, polylactide-co-glycolide, polyethylenglycol, gentamicin sulfate, antimicrobial effect

## Abstract

Nanotechnology is a promising approach both for restoring or enhancing activity of old and conventional antimicrobial agents and for treating intracellular infections by providing intracellular targeting and sustained release of drug inside infected cells. The present paper introduces a formulation study of gentamicin loaded biodegradable nanoparticles (Nps). Solid-oil-in water technique was studied for gentamicin sulfate nanoencapsulation using uncapped Polylactide-co-glycolide (PLGA-H) and Polylactide-co-glycolide-co-Polyethylenglycol (PLGA-PEG) blends. Screening design was applied to optimize: drug payload, Nps size and size distribution, stability and resuspendability after freeze-drying. PLGA-PEG concentration resulted most significant factor influencing particles size and drug content (DC): 8 *w*/*w*% DC and 200 nm Nps were obtained. Stirring rate resulted most influencing factor for size distribution (PDI): 700 rpm permitted to obtain homogeneous Nps dispersion (PDI = 1). Further experimental parameters investigated, by 2^3^ screening design, were: polymer blend composition (PLGA-PEG and PLGA-H), Polyvinylalcohol (PVA) and methanol concentrations into aqueous phase. Drug content was increased to 10.5 *w*/*w*%. Nanoparticle lyophilization was studied adding cryoprotectants, polyvinypirrolidone K17 and K32, and sodiumcarboxymetylcellulose. Freeze-drying protocol was optimized by a mixture design. A freeze-dried Nps powder free resuspendable with stable Nps size and payload, was developed. The powder was tested on clinic bacterial isolates demonstrating that after encapsulation, gentamicin sulfate kept its activity.

## 1. Introduction

Gentamicin is an aminoglycoside antibiotic used to treat several types of bacterial infections, including bone infections, endocarditis, pelvic inflammatory disease, meningitis, pneumonia, urinary tract infections and sepsis. Moreover, it is the preferred antibiotic to treat nosocomial infections caused by bacteria such as *E. coli*, *Pseudomonas aeruginosa* and *Staphylococcus aureus*. 

It is a small drug molecule (Mw 477.596 g/mol); classified as BCS (biopharmaceutical classification system) class III because of its high water solubility and poor cellular penetration. Gentamicin mechanism of action involves irreversible binding to 30S ribosomal subunit, inhibition of messenger RNA (mRNA) complex formation leading to protein synthesis prevention and resulting in cell bacteria death. Additionally, as all aminoglycoside antibiotics, gentamicin can cause membrane damage altering ionic concentration [1]. The conventional multiple dosing regimens result in adverse reactions due to fast gentamicin clearance, or its unfavorable biodistribution, causing nephrotoxicity and ototoxicity.

A biodegradable nanoparticulate drug delivery system (Nps DDS) loaded with gentamcin can be a promising strategy to reduce gentamicin side effects meanwhile prolonging its activity. Gentamicin loaded Nps can provide an appropriate drug release kinetic supplying an effective and efficacious local therapeutic concentration of antibiotic at infection site [2,3]. Moreover, Nps DDS could demonstrate some advantages in treating biofilm formation, improving antimicrobial activity over than effectiveness and safety antibiotic administration, as reported in the literature [4,5,6].

Nanotechnology has emerged as a promising approach both for restoring or enhancing activity of old and conventional antimicrobial agents and for treating intracellular infections by providing intracellular targeting and sustained release of drug inside infected cells. Nps may lead to an improvement in drug cellular accumulation and a reduction of the required dosing frequency improving patient compliance and efficacy of antimicrobial therapy. They represent a promising strategy to overcome microbial resistance [4,7].

According to their sub-micro size, nanoparticles efficiently cross biological barrier improving drug bioavailability and permanence time at infected site, protecting drug from degradation and achieving gradual release pattern. In this context, loading gentamicin in polymeric nanoparticles could be interesting in reducing antibiotic resistance and adverse effect, improving treatment of infections [5,8,9,10].

According to literature, several authors studied the preparation of gentamicin loaded nanoparticles based on PLGA using different method as water in oil in water (w/o/w) and solid in oil in water (s/o/w) evaporation techniques [5,11,12].

Nevertheless, no publication to our knowledge investigated PLGA-PEG/PLGA-H blends in the preparation of nanoparticles by s/o/w extraction method. The aim of the present work was to set up a suitable preparation method in order to obtain stable PLGA-PEG/PLGA nanoparticles with high gentamicin sulfate payloads. It is known from the literature that drug payloads represent an issue in Nps, especially when the drug needs to be administered in high doses [13]. As previously reported, gentamicin sulfate is a BCS class III drug with high water solubility and small Mw, this makes its encapsulation challenging. The experimental work was approached in three phases. Firstly, nanoparticles were prepared by s/o/w method and characterized with regard to size, size distribution, drug content and drug release. In the first part of the work, two different full factorial screening experimental designs were used in order to optimize process-parameters. The effect of several process parameters was investigated in order to reduce particle size and to enhance drug encapsulation. In vitro release tests were performed on optimized formulations in phosphate buffer saline (PBS) pH 7.4, 37 °C in dynamic conditions. Gentamicin release kinetic from the Nps was evaluated by fitting drug release data with four kinetic equations: zero order, first order, Higuchi model and Korsmeyer-Peppas models. A design of experiment (DoE) approach was adopted to investigate the influence of all process parameters, to evaluate interactions between process variables, and to methodically control them during nanoparticle synthesis. Second phase of the work dealt with formulation study. Gentamicin sulfate Nps are lyophilized to get a stable powder. Lyophilization is a process frequently used in the pharmaceutical industry in order to achieve a medicinal product with suitable stability for the required product shelf life. The process requires addition of cryoprotectant agents in order to get a freely resuspendable powder. Aspects such as resuspendability and Nps stability upon Nps powder reconstitution are fundamental for a medicinal product. A freeze-drying protocol and cryoprotectant agent selection were optimized by using a mixture design. A freeze-dried powder, able to maintain nanoparticles resuspendability, and their stability as long as size and payload is concerned, was developed. 

Eventually, the third phase of the work dealt with evaluation of antimicrobial activity of gentamicin sulfate loaded Nps. The tests were conducted against five different Gram-positive and Gram-negative bacteria from clinic bacterial isolates such as *Proteus mirabilis*, *Pseudomonas aeruginosa* and *Staphyloccocus aureus*. Standard *E. coli* ATCC 25922 was used as control. The investigation plan was organized in order to get wide information on gentamicin sulfate loaded Nps activity against bacterial strain commonly involved in severe infectious diseases, and in not standardized conditions. 

## 2. Materials and Methods

### 2.1. Materials

Gentamicin sulfate (Gentamicin C1 C_21_H_43_N_5_O_7_, Mw 477.6 g/mol, Gentamicin C2 C_20_H_41_N_5_O_7_, Mw 463.6 g/mol, Gentamicin C1a C_19_H_39_N_5_O_7_, Mw 449.5 g/mol was from Sigma-Aldrich (Sigma-Aldrich, Milano, Italy). Uncapped polylactide-co-glycolide (PLGA-H, 7525 DLG 3A Mw 25 kDa) and polylactide-co-glycolide-co-polyethylenglycol (PLGA-PEG, 5050 DLG 5C PEG 1500 Mw 70 kDa, PEG 51%) were from Lakeshore Biomaterials, Birmingham, AL, USA. PVA (Mw 85–124 kDa 87–89% hydrolyzed), polyvinylpirrolidone (PVP K17, Mw 17 KDa and PVP K32, Mw 32 KDa), sodium carboxymethylcellulose (SCM, Mw 90 KDa) methanol, ethanol, acetone, dichloromethane, dimethyl sulfoxide, ninhydrin, Mw 178.14 g/mol were from Sigma Aldrich, Milano, Italy.

### 2.2. Preparation of Nanoparticles

Nanoparticles were prepared using a modified solid/oil/water extraction method (s/o/w). Briefly, 3.5 mg of gentamicin sulfate was dissolved in 0.1 mL of distilled water. The gentamicin sulfate solution was then added to 2 mL of acetone containing different amounts of polymer (50 or 25 mg). The diffusion of water into acetone contributes to gentamicin sulfate precipitation. The suspension was stirred by vortex at 30,000 rpm for 1 min and then added to different volumes of PVA solutions at 1 *w*/*v*% (10 or 5 mL). Following acetone phase diffusion into the aqueous PVA phase contributed to the formation of gentamicin sulfate-loaded nanoparticles.

### 2.3. Optimization Protocol by Experimental of Design (DoE)

S/o/w technique was submitted to a screening design (2^3^) to investigate: (a) effect of polymer concentration (mg/mL); (b) volumetric ratio between solvent (S, acetone) and non-solvent (nS, PVA aqueous solution) and (c) stirring rate (rpm), keeping polymer (2 mL) volume constant. These factors (input) were selected because they strongly influenced particle size (nm), size distribution (PDI) and drug content (µg of gentamicin entrapped/mg of nanoparticles). Eight batches were prepared for 2^3^ full factorial design to study the effect of the three independent variables (input) on each response (output). Each factor was tested at two level designed as −1 and +1, as reported in Table 1.

Equation (1) is:

Y = β_0_ + β_1_X_1_+ β_2_X_2_ + β_3_X_3_ + β_12_X_1_X_2_ + β_13_X_1_X_3_ + β_23_X_2_X_3_ (1)

Intercept = β_0_

Linear terms = β_1_X_1_+ β_2_X_2_ + β_3_X_3_

Interaction terms = β_12_X_1_X_2_ + β_13_X_1_X_3_ + β_23_X_2_X_3_

The coefficients corresponding to linear effects (β_1_, β_2_ and β_3_) and to interactions (β_12_, β_13_, and β_23_) were determined from the results of all experiments in order to identify a statistically significant term. Diagrammatic representation of values per each response (pareto chart and response surface) resulted to be very helpful to explain the relationship between independent and dependent variables. 

After this screening design, other factors such as: (a) type of polymer solvent; (b) polymer composition; (c) PVA concentration in the outer phase; (d) addition of methanol and ethanol in PVA outer phase, were investigated in order to improve gentamicin sulfate content. A second 2^3^ full factorial design was performed using Statgraphics Centurion software (Table 2, Statgraphics Centurion software distributed by software online distribution University of Pavia, Pavia, Italy) and it was designed based on the preliminary experimental results reported in results and discussion. Eight batches were prepared for the 2^3^ full factorial design, keeping constant the polymer concentration (12.5 mg/mL), solvent/non solvent ratio (0.5 *v*/*v*) and stirring rate (700 rpm) as already set up from the first screening design. 

This second study was assessed in order to optimize the effect of: (a) polymer composition (PLGA-PEG and PLGA-H); (b) PVA concentration (*w*/*v*%) and (c) addition of a different percentage of a non-solvent (MetOH) into PVA outer solution, on three responses as gentamicin sulfate content (drug content, DC), particles size and size distribution. Each factor was tested at two levels designated as −1, and +1 (Table 2). This second experimental screening design was planned to improve gentamicin drug content keeping constant values of particle size and particle size distribution obtained from the first screening design. The regression equation for the response was calculated using Equation (2):

Y = β_0_ + β_4_X_4_+ β_5_X_5_+ β_6_X_6_ + β_45_X_4_X_5_ + β_46_X_4_X_6_ + β_56_X_5_X_6_ (2)

Response in the above equation Y is the measured response associated with each factor level combination: βo is the intercept, β is the coefficient of terms X, X_4_, X_5_ and X_6_, which are the studied factors; X_4_X_5_, X_4_X_6_ and X_5_X_6_ are the interaction between variables. 

Response surface and pareto charts methodology set a mathematical trend in the experimental design for determining the influence of each experimental factor and their interactions for a given response.

Two replications were run for each screening design.

### 2.4. Redispersability and Lyophilization Study of Nanoparticles

The nanoparticle composition selected from the two screening designs was batch Np 13 (see Table 2). batch Np 13 was purified by centrifugation at 16,400 rpm, 4 °C for 20 min. The suspension was frozen at −25 °C for 1 h and then −40 °C for 12 h and then lyophilized at −48 °C at 0.01 mbar for 24 h (Freeze drying apparatus, LIO 5P, Milan, Italy). Freeze−drying can generate many stresses, it can induce aggregation and in some cases irreversible nanoparticles fusion. For this reason, cryoprotectant solution must be added to the suspension of nanoparticles before freeze drying, in order to protect these fragile systems. Polyvinylpirrolidone (PVP K17 and/or PVP K32) and sodium carboxymethylcellulose (SMC) were chosen as cryoprotectants in order to obtain a 1:2 weight ratio between nanoparticles and cryoprotectant. The lyophilized nanoparticles formulation in the presence of cryoprotectant was rehydrated in 500 µL of sterile water (same volume of starting cryoprotectant solution). In order to investigate the influence of cryoprotectant as such or their mixture a Mixture Design experimental approach was applied. The simplex centroid (centroid) mixture design was selected for the study; it includes in 2q-1 different blends design (q number of components) generated from the processing of: pure components (1,0,0), binary mixtures (1/2, 1/2, 0) and ternary mixtures (1/3, 1/3, 1/3) until reaching the selected centroid (1/q, 1/q, 1/q), in our case the centroid corresponds to the ternary mixture (see Table 3). The three components of the mixture are: (i) polyvinylpyrrolidone PVP K17 (ii) polyvinylpyrrolidone PVP K32 and (iii) sodium carboxymethyl cellulose (SCM). In the case of binary, each component of the mixture must correspond to 100% and for the ternary mixtures, each component is 66.6%. The particle size was determined before and after freeze-drying, and ratio between final and initial size (S_f_/S_i_) was calculated. 

### 2.5. Particle Size and Surface Charge Analysis

Particle size and polydispersity index (PDI) were determined by dynamic light scattering (DLS) with ZetaSizer (NICOMP 380 ZLS, Particles Sizing System, Santa Barbara, CA, USA). Each fresh formulation was dispersed in distilled water and appropriately diluted reaching a concentration of 13 µg/mL. Zeta potential was evaluated using ZetaSizer (NICOMP 380 ZLS, Particles Sizing System, Santa Barbara, CA, USA). Each fresh formulation was dispersed in PBS (10 mM) at concentration of 13 µg/mL. All measurements have been carried out in triplicate.

### 2.6. Morphology

Shape and surface morphology of nanoparticle formulation were examined with a transmission electron microscopy (TEM) (TEM 208 S, Philips NL, Eindhoven, The Netherlands). 15 µL of Nps suspension was placed on a 300 mesh copper grid covered with Formvar film (AGAR Scientific, Stansed, UK). The excess liquid was removed with filter paper, and then 10 μL of 1% uranyl acetate was added on to grids and left standing for 10 s, after that, liquid in excess was removed by filter paper and sample analyzed. 

### 2.7. Drug Content Determination

13 mg of Nps (the weight corresponds to one batch size) was dispersed into 1 mL of dimethyl sulfoxide (DMSO), the dispersion has stirred at 300 rpm for 5 h to ensure complete dissolution of nanoparticles. The resulting solution was centrifuged at 16,400 rpm, 25 °C for 20 min and pellet was reconstituted in 2 mL of distilled water and stirred for 12 h to solubilize the extracted gentamicin. Both supernatants, in DMSO and in distilled water, containing gentamicin sulfate were analyzed by ultraviolet-visible (UV-Vis) spectroscopy at λ 400 nm after reaction with ninhydrin [9,10]. In regards to ninhydrin assay: 800 μL of supernatant was mixed with a ninhydrin solution in PBS pH 7.4 (240 μL, 0.2 *w*/*v*%), the mixture was vortexed and heated in a water-bath at 95 °C for 15 min, and then cooled in an ice-bath for 10 min.

A calibration curve in DMSO (50–500 µg/mL, R^2^ = 0.9879) and a calibration curve in water (50–500 µg/mL, R^2^ = 0.9909) were used for gentamicin sulfaate quantification. Drug Content (DC) was calculated using Equation (3), considering the contribution from DMSO and distilled water:

(3) $$\mathrm{DC}=\frac{weight\;of\;gentamicin\;extracted\;(\text{µ}g)}{weight\;of\;dried\;nanoparticles\;(mg)}\times 100$$

### 2.8. In Vitro Release Study

In vitro release study on gentamicin sulfate-loaded nanoparticles was performed as follows: 90 mg of lyophilized nanoparticles formulation composed by 30 mg of gentamicin sulfate loaded Nps and 60 mg of cryoprotectant (the formulation selected from DoE mixture study), were suspended in 1 mL of PBS pH 7.4, at 37 °C. At each time point (0.25, 0.5, 1, 2, 4, 6, 8, 10 h), nanoparticles were centrifuged (20 min, 25 °C at 16,400 rpm) and 800 µL of incubation medium (PBS) collected and replaced by an equal amount of fresh PBS. The amount of gentamicin sulfate released at each time point was detected by reaction with ninhidryn and then quantified by ultraviolet-visibile (UV-Vis) spectrophotometer (UV-1601, Shimadzu, Japan) at 400 nm using a calibration curve in PBS (33.3–275 µg/mL, R^2^ = 0.9979). The release study was conducted until to reach 100% of release. Gentamicin sulfate as such (1 mg) underwent a dissolution test in the same experimental conditions. All experiments were performed in triplicate. Four kinetic models were applied to analyze the in vitro drug release data for release kinetics fitting.

The zero order (Equation (4)) explains the release from systems where rate of drug release is concentration independent [14]

(4) $$C\;={\;K}_{0t}t$$

where *C* is the concentration of drug at time *t*, *t* is the time and K_0_ is zero-order rate constant express in concentration/time unit.

The first order (Equation (5)) explains the release from systems where rate of drug release is concentration dependent.

(5) $$\;\log C_{0}-\;\log C\;={\;K}_{1}t/2.303$$

where *C*_0_ is the initial concentration of drug and K_1_ is the first order rate constant.

Higuchi model describes the release from insoluble matrix as square root of time dependent process based on Fickian diffusion as in Equation (6) [14].

(6) $$C\;={\;K}_{h}\sqrt{t}$$

where, K_h_ is the constant which reflects system design variables.

Korsmeyer-Peppas model describes the release of drug from a polymeric system (Equation (7)).

(7) $$M_{t}/M_{\infty }={\mathrm{KK}}_{\mathrm{hp}}t^{n}$$

where *M_t_*/*M* is the fraction of drug released at time *t*, K_hp_ is the rate constant and *n* is the release exponent.

### 2.9. In Vitro Gentamicin Activity Determination Against Clinical Isolates

In order to evaluate the antibacterial activity of gentamicin and gentamicin-loaded Nps, the broth micro-dilution method was carried out against five different Gram-positive and Gram-negative bacteria. MIC (Minimum Inhibitory Concentration) and MBC (Minimum Bactericidal Concentration) tests were carried out. The three Gram-negative clinical strains tested were *Proteus mirabilis* (Gentamicin MIC = 4 mg/L; Gentamicin MBC = 8 mg/L), *Escherichia coli* (Gentamicin MIC = 2 mg/L; Gentamicin MBC = 4 mg/L) and *Pseudomonas aeruginosa* (Gentamicin MIC = 1 mg/L; MBC = 2 mg/L). The two remaining Gram-positive clinical strains tested were the *Staphyloccocus aureus* 695 (Gentamicin MIC = MBC = 1 mg/L) and the *S. aureus* 728 (Gentamicin MIC = 8 mg/L; GN MBC = 16 mg/L). An *Escherichia coli* ATCC 25922 (Gentamicin MIC = MBC = 0.5–2 mg/L) was used as quality control in each in vitro test.

Gentamicin sulfate was sterilized by filtration using 0.22 μm Millipore membranes.

The MIC and MBC in vitro values determinations were performed with the aim to preliminary evaluate antibacterial activity of gentamicin sulfate Nps. The test is useful in order to define: (i) if gentamicin maintains its activity and/or increases it, after encapsulation; (ii) the quantity of gentamicin loaded Nps to be administered. 

A stock concentration of free drug and of gentamicin sulfate-loaded Nps was prepared in deionized water that was further diluted in Mueller Hinton (MH) broth to reach a concentration range of 0.06 to 16 mg/L for Gram negative organisms and between 0.06 nd 32 mg/L in the case of Gram positive bacteria. The final concentration of bacteria in the individual tubes was adjusted to about 5 × 10^5^ colony-forming unit (CFU)/mL.

**Figure 1:**
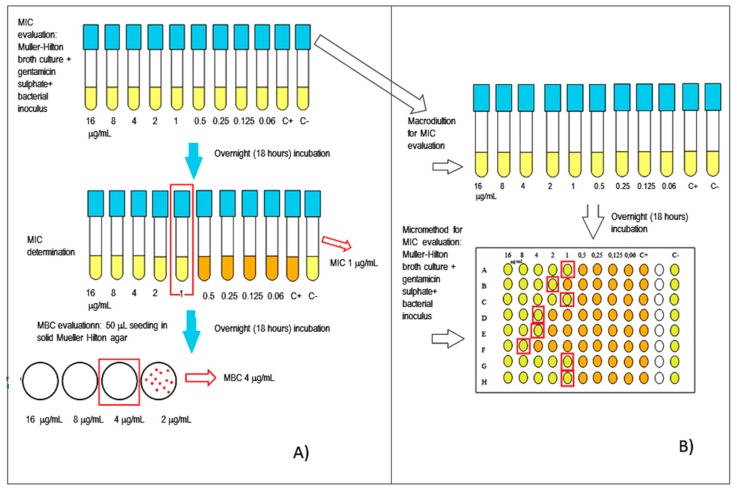
Schemes of: (**A**) MIC and MBC tests; (**B**) MIC test by micro-method.

After 24/48 h of incubation at 37 °C, the test tubes were examined for possible bacterial turbidity, and the MIC of each test compound was determined as the lowest concentration that could inhibit visible bacterial growth. After MIC determination, an aliquot of 10 µL from all tubes in which no visible bacterial growth was observed was seeded in Mueller Hinton agar plates. The plates were then incubated for 48 h at 37 °C. The MBC endpoint is defined as the lowest concentration of antimicrobial agent that kills 99.9% of the initial bacterial population where no visible growth of the bacteria was observed on the plates, following the European Committee on Antimicrobial Susceptibility Testing [15]. Figure 1 reports a scheme showing how the tests were conducted. Experiments were performed in triplicate.

To verifiy bacterial growth, an aliquot of 10 µL for each bacterial strain was withdrawn from the tube containing the highest Nps suspension concentration and seeded in agar plates (10 cm diameter). The agar plates were incubated overnight at 37 °C and analyzed. 

### 2.10. Bacterial Survival Test

The bacterial survival test was performed on both *E. coli* ATCC 25922 quality control and all the Gram positive and Gram negative isolates included in the study. Aim of the test was to evaluate if the nanoparticles concentrations added, performing the MIC and MBC tests (or higher) could somehow affect growth and/or vitality of the above mentioned microorganisms. 

Four different nanoparticle concentrations of 142, 2.8, 4.99 and 5.7 mg/mL were tested for bacterial survival in MH broth. The bacterial inocula were of 5 × 10^4^ CFU/mL.

The results were recorded by visual inspection of the tubes after 18 h of incubation at a temperature (T) of 35 °C + 2 °C.

An aliquot of 10 µL was than collected from each tube- and seeded in MH agar plates; after overnight incubation at 35 ± 2 °C, bacterial growth was recorded (Figure 1).

### 2.11. Statistical Analysis

All experiments were based on three independent samples and the experiments were repeated for three times. Results are reported as mean ± standard deviation. Moreover, analysis of variance (ANOVA) and *p*-value < 0.05 were used to assess statistical significance.

## 3. Results and Discussion

Physical properties, as particles size, size distribution and drug content (DC) are summarized in Table 4. All PLGA-PEG nanoparticles were prepared using a s/o/w procedure, several process parameters were evaluated to optimize size, size distribution and DC.

Keeping constant PLGA-PEG concentration at 12.5 mg/mL, and stirring rate at 350 rpm, increase of S/nS ratio causes an important increment of size and size distribution (Batch #1 and 2). As reported in literature, S/nS ratio is a critical parameter having an important role in nanoparticle formation [16]. The results did not highlight statistical differences in term of drug content that can be attributed S/nS ratio variation.

The increment of stirring rate up to 700 rpm leads to reduction of nanoparticles size (240.0 ± 0.54 nm) and size distribution values (PDI: 0.130 ± 0.57). A slight increase of drug content value was observed, reaching 4.38 ± 2.45 µg gentamicin sulphate/mg nanoparticles. Low drug content values can be due both to the high drug solubility in aqueous medium (50 mg/mL) and the large nanoparticles surface area, which facilitate gentamicin sulfate diffusion into external aqueous phase during nanoparticles preparation process. Nanoparticles prepared with polymer concentration of 25 mg/mL show size >500 nm. Only for batch #7 no statistical variations of size were detected; nevertheless, polydispersity index (PDI) was larger (1.230 ± 0.24). In terms of drug content, high polymer concentration does not affect gentamicin sulfate entrapment.

From data reported in Table 4 polymer concentration, S/nS ratio and stirring rate were selected as the most critical process parameters for the preparation of gentamicin sulfate-loaded nanoparticles. These parameters were further studied in order to increase gentamicin content in PLGA-PEG nanoparticles.

The data reported in Table 4 were applied for a 2^3^ randomized screening design (DoE), the three factors were evaluated at two different levels, summarizing all possible combinations.

PLGA-PEG polymer concentration (25–12.5 mg/mL), S/nS ratio (0.5–0.2 *v*/*v*) and stirring rate (700–350 rpm) were chosen as independent variables whereas particle size, size distribution and drug content were selected as dependent variables (outputs). Terms with *p* < 0.05 were considered statistically significant and retained in the reduced model.

**Figure 2:**
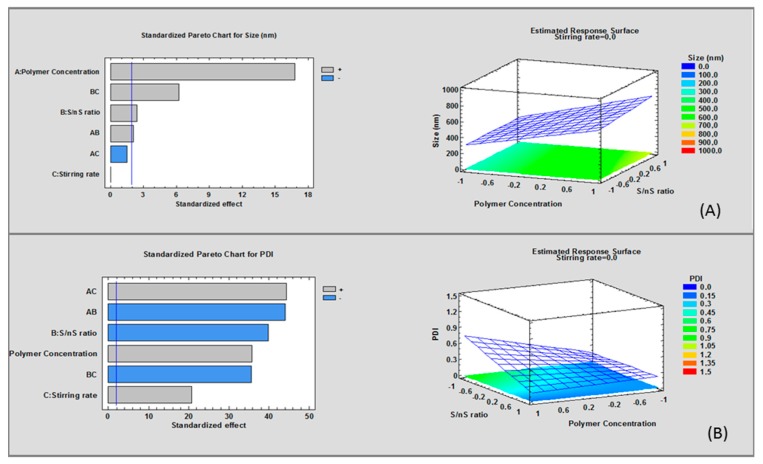
Estimated response: (**A**) surface and pareto chart for particle size; (**B**) size distribution of the screening design.

The Pareto Chart and Estimated Response Surface of mean diameter versus polymer concentration and S/nS ratio (significant factors, Figure 2A) show a linear model. Namely, nanoparticles size is linearly dependent to polymer concentration and S/nS ratio. Higher particles size values were observed at high polymer concentration and at high values of S/nS ratio.

The coefficients over the blue line (significant limit) having *p*-value < 0.05 are highly significant (Pareto Chart Figure 2B). The interaction BC, between factor B (S/nS *v*/*v* ratio) and factor C (stirring rate) shows *p* value < 0.05, thus it contributes on an increase of mean particle size. Nevertheless, factor C does not have any significant effect on mean diameter. Equation of the full model is here reported:

Size (nm) = 487.313 + 178.637 × polymer concentration (*w*/*v%*) + 25.5375 × S/nS ratio + 0.4875 × stirring rate (rpm) + 21.9125 × polymer concentration (*w*/*v%*) × S/nS ratio − 16.1875 × Polymer concentration (*w*/*v%*) × stirring rate + 66.1625 × S/nS ratio × stirring rate (rpm).

R^2^ squared is a measure of total variability explained by the model. R^2^ squared value of the model was 61.10 indicating that the model can explain 61.10 of variability around the mean. 

The pareto chart of the model shows that polymer concentration and S/nS ratio significantly (*p* < 0.05) influence output, that is mean particle size. Particle size values were significantly bigger (507.8 ± 47.9–919.3 ± 53.2 nm) for formulations with high polymer concentration (25 mg/mL) and high S/nS *v*/*v* ratio (0.5).

**Figure 3:**
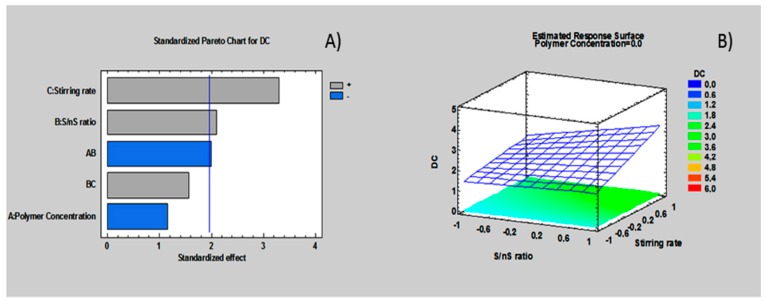
Estimated response: size distribution DC of the screening design: (**A**) standardized pareto chart; (**B**) estimated response surface polymer concentration.

A value of PDI close to 0 indicates homogeneous dispersion, while PDI values higher than 0.3 represent heterogeneous distribution. Low PDI was measured at low values of both polymer concentration and stirring rate and at high value of S/nS ratio (Figure 3). 

The pareto chart of the full model for PDI shows that all factors were significant (*p*-value < 0.05) contributing in output prediction (PDI), (Figure 3). Full model analysis of variance (ANOVA) showed a R^2^ square value of 87.42, the equation is reported here below:

PDI = 0.327 + 0.12675 × Polymer concentration (*w*/*v%*) − 0.14075 × S/nS ratio + 0.0735 × Stirring rate − 0.156 × Polymer concentration (*w*/*v%*) S/nS ratio + 0.15675 × Polymer concentration (*w*/*v%*) stirring rate − 0.12575 × S/nS ratio × stirring rate (rpm)

PDI is lower for high value of S/ns *v*/*v* ratio (0.5), indicating a narrow particle size distribution of nanoparticles, instead for high polymer concentration (25 mg/mL) and high stirring rate (700 rpm) nanoparticles suspension is non uniform with some aggregation phenomena. 

As observed for mean particle size and PDI outputs, DC response surface shows a linear model (Figure 2A), thus no further experiment was designed. High value of S/nS *v*/*v* ratio and stirring rate are shown to improve DC (Figure 2B and Figure 3).

ANOVA of reduced model show a R^2^ squared value of 84.07. The reduced model has the following equation:

DC (μg/mg Nps) = 2.12875 − 0.20375 × Polymer concentration (*w*/*v%*) + 0.37125 × S/nS ratio + 0.5875 × stirring rate (rpm) − 0.35125 × Polymer concentration (*w*/*v%*)× S/nS ratio

DC pareto chart indicates that stirring rate and S/nS *v*/*v* ratio have significantly important influence. 

This preliminary screening design demonstrates that: (i) polymer concentration at low level (12.5 mg/mL) contributes to reduce both particles size and PDI; (ii) S/nS *v*/*v* ratio at high level (0.5) positively influences DC and PDI values, and it negatively affects mean particle size; (iii) stirring rate is the most important factor affecting DC, in such a way the highest DC value was measured at high stirring rate value. On the basis of the preliminary results summarized in Table 4, and of the statistical values of DoE, process parameters corresponding to batch #4 were selected. Nevertheless, further attempts were performed to improve gentamicin sulfate DC. Effect of several processes variables was evaluated, such as solvent used to dissolve polymer, polymer composition and composition of external aqueous phase with regard to PVA concentration, and addition of an alcohol. The effect of all process parameters was investigated on DC, particle size and particle size distribution (Table 5).

### 3.1. Effect of Solvent

Solvents used to dissolve polymer have an important role in the preparation of Nps, because they affect both size and DC. Solvent diffusion into the outer phase should be fast enough to permit polymer precipitation and drug entrapment inside nanoparticles. It is important to evaluate solvent affinity for external aqueous phase (not solvent) in order to control solvent diffusion towards the aqueous phase [17]. The solvents properties evaluated are: solvent power towards the polymer and dielectric constants. The latter property provides a measure of solvent polarity and it can be an acceptable predictor of solvent ability to dissolve ionic compounds. 

Acetone and DMSO were selected for preparing of PLGA-PEG Nps containing gentamicin sulfate. Acetone is the most common solvent used in s/o/w technique because it is a good solvent for PLGA polymer and it has a low dielectric constant (ε = 20.5 at 25 °C), while DMSO is a polar solvent with high dielectric constant (ε = 46.4 at 25 °C). DMSO shows low affinity for PVA aqueous phase hampering solvent diffusion from polymer matrix to the outer phase, while the high affinity of acetone with PVA aqueous solution should facilitate diffusion of polymer solvent into external aqueous phase reducing time needed for polymer precipitation and potentially increasing drug entrapment. 

Batches #4 and 9 were prepared with acetone and DMSO, respectively. On the basis of data reported in Table 5, acetone remains the optimal solvent for preparing PLGA-PEG Nps using s/o/w technique. Indeed, the formulation obtained solubilizing PLGA-PEG polymer in DMSO (Batch # 9) show particles size >1 µm, due to aggregates formation during polymer precipitation. 

### 3.2. Effect of Polymers

The effect of polymer composition was also investigated using PLGA copolymer and PLGA-PEG block copolymer. DC resulted to depend on polymer composition: drug content obtained for Batch #10 (27.31 µg/mg of nanoparticles) is 8 times higher with respect to n that of Batch #4. It can be hypothesized that ionic interaction between carboxyl groups of PLGA-H and amino groups of gentamicin sulfate led to improve drug entrapment efficiency.

PLGA-PEG and PLGA-H polymer were mixed at different ratio (70:30, 50:50, 30:70). As expected, the reduction of PLGA-H% in organic phase causes an important decrease of DC reaching 0.2 µg of gentamicin per mg of nanoparticles. Batches obtained mixing PLGA-H with PLGA-PEG show higher particles size and size distribution compared with Batches #4 and 10. Only Batch #12 (PLGA-PEG/PLGA-H ratio, 50:50) shows similar results in terms of size and size distribution, nevertheless low DC value was measured (0.94 µg/mg of nanoparticles). The ratio PLGA-PEG/PLGA-H 30:70 (Batch #11) was selected and further parameters were optimized changing PVA concentration and adding an alcohol into external aqueous phase.

### 3.3. Effect of PVA Concentration and Addition of Alcohol into External Aqueous Phase

PVA concentration affects external aqueous phase viscosity and consequently acetone diffusion rate from polymer matrix to external aqueous phase: external aqueous phase viscosity is reduced decreasing PVA concentration promoting solvent diffusion. The rapid diffusion of polymer solvent promotes drug entrapment into polymer matrix and facilitates small nanoparticles formation. On the opposite, lower external aqueous phase viscosity facilitates gentamicin sulfate diffusion from the embryonic nanoparticles into the aqueous outer phase. Therefore an equilibrium should be reached between the two competitive effects, maximizing drug loading and minimizing particle size. Results of Batches #14 and #15 demonstrate gentamicin sulfate diffusion into the external aqueous phase prevails. In fact, at low PVA percentages (0.5 and 0.25 *w*/*v*%), DC values were lower with respect to Batch #11 and particles size value was very high. The concomitant reduction of PVA concentration and addition of alcohols into external aqueous phase was investigated. Alcohols are characterized by low dielectric constant which affects gentamicin sulfate solubility and its capability to escape into external aqueous phase. Two different alcohols were evaluated: ethanol (ε = 24.6 at 25 °C) and methanol (ε = 32.7 at 25 °C). Addition of alcohol to the external aqueous phase should reduce PVA aqueous phase dielectric constant increasing DC values. Batch #16 and #17 were prepared using low PVA solution concentrations (0.5 and 0.25 *w*/*v*%) and adding 30 *v*/*v*% of ethanol. The results show an important significance on DC which is dependent on ethanol addition despite PVA concentration. Nevertheless, nanoparticles size increases reaching 1 µm.

Different percentages of ethanol (20, 30, 40 and 60 *v*/*v*%) were tested maintaining constant PVA percentage at 0.25 *w*/*v*% (Batches #17–20). The best results regarding particles size and DC were obtained for batch #17.

Batch #21 was prepared using same process parameters of batch #17, but MetOH has used instead of EtOH. Addition of 30 *v*/*v*% of MetOH into the external aqueous phase allows to increase DC up to 12 µg/mg nanoparticles keeping Nps size at 310 ± 111 nm. The effect cannot be explained by MetOH dielectric constant, being higher than EtOH dielectric constant. However, PLGA-PEG/PLGA-H polymer blend has slight higher affinity for EtOH compared to MetOH, and this can slow down Nps precipitation with consequent increase of gentamicin sulfate diffusion. 

In conclusion, polymer composition, PVA concentration (*w*/*v*%) and addition of MetOH into the aqueous phase were the most significant variables influencing DC and size of Nps. 

This preliminary study (Table 5) using an empirical approach was enhanced through a full factorial experimental design in order to statistically evaluate the selected variables and to investigate their interaction. The interactions among polymer composition, PVA concentration and methanol addition were examined using a 2^3^ full factorial design by Statgraphic centurion Software. Polymer compositions (PLGA-PEG/PLGA-H ratios 70:30 and 30:70), PVA and methanol concentrations (0.25, 0.5 *w*/*v*% and 30, 60 *v*/*v*% respectively) were defined as inputs, while size (nm) size distribution and DC (µg/mgNp) were the outputs. Table 6 summarizes run parameters and responses for 2^3^ (three factors at two levels) random screening design. Data analysis from pareto chart show that PVA concentration and the addition of MetOH to PVA aqueous solution have a significant (*p*-value < 0.05) impact on the DC. In particular, formulations with high PVA concentration (0.5 *w*/*v*%) and high percentage of MetOH added to PVA solution (60 *v*/*v*%) result in lower DC but their interaction, although is not so significant, has a positive influence on DC. The response surface show a linear model in which the DC highest value should be obtained for the composition with 0.25 *w*/*v*% PVA and 30 *v*/*v*% of MetOH (see Supplementary Materials Figures S1 and S2). 

PDI results from pareto chart (see Figure S2 Supplementary Material) indicate that only PVA *w*/*v*% concentration positively influences on the response. Low PDI value was detected at lowest PVA concentration (0.25 *w*/*v*%). The response surface of PVA *w*/*v*% versus polymer composition shows low PDI value indicating homogeneous suspension for PLGA-PEG/PLGA-H 30/70 ratio and PVA concentration 0.25 *w*/*v*%. The equation based on the statistical reduced model (R^2^-squared = 96.05%) is:

PDI = 0.266 − 0.1185 × Polymer composition + 0.259 × PVA (*w*/*v%*) + 0.03 × MetOH (*w*/*v*%) − 0.164 × Polymer composition × PVA (*w*/*v*%) + 0.195 × Polymer composition × MetOH (*w*/*v*%).

Pareto chart analysis (see Figure S2 Supplementary Material) shows that both methanol addition and the interaction between methanol and PVA *w*/*v*% concentration have a significant effect on particle size. Particles with size >500 nm were obtained at high value of factors C, which is MetOH at high level (60 *v*/*v*%). Moreover, PVA concentration (*w*/*v*%) does not have a significant influence on the response (size), but the interaction between PVA concentration (*w*/*v*%) and MetOH addition in PVA external solution has a significant impact on particle size. Smallest particles size (nm) is obtained with the addition of low percentage of MetOH (30 *w*/*v*%) at lower PVA concentration of 0.25 *w*/*v*%, as it shown by the response surface for particle size (nm). The equation based on this statistical design (R^2^ squared = 87.29%) of the reduced model were reported:

Size (nm) = 356.95 − 38.4 × Polymer composition + 259.7 × PVA (*w*/*v*%) + 456.25 × MetOH (*v*/*v%*)− 488.7 × PVA (*w*/*v*%) × MetOH (*v*/*v%*). 

In conclusion, nanoparticles size and DC depend on methanol addition into external aqueous phase and PVA polymer concentration, while PDI is a result of polymer composition and PVA concentration. On the basis of this second screening full factorial design, Batch #25 was selected for a further deeper investigation on stability after freeze-drying, morphology, and gentamicin sulfate in vitro release test. 

After the optimization study by DoE, Batch #25 was purified by centrifugation and suspended in distilled water. Several experimental conditions, during Nps preparation and Nps recovery, were optimized (Table 7), it was demonstrated that prolonging curing time from 4 to 5 h, it is possible to limit aggregation phenomena after recovering by centrifugation (condition B, Table 7). Moreover, pellet resuspension requires a gradual addition of water and cycles of vigorous stirring by vortex and sonication. The different resuspension and curing conditions did not affect DC. 

As reported in Table 7 Batch #25, selected on the base of the results of optimization study, was suspended in 12 min following resuspension conditions C.

The results in Table 8 show that PVP K17 and K32 seem to stabilize the nanoparticles during freeze-drying: S_f_/S_i_ ratio values are 1 and 1.19, respectively, confirming that there aren’t aggregation phenomena. All formulations containing cryoprotectants show good aspect after lyophilization with no evidence of collapse phenomena with the exception freeze dried formulation #3 (see Table 8). This is probably due to high viscosity of SCM solution that limits re-hydration of the lyophilized nanoparticles. Samples containing SCM show S_f_/S_i_ values >1.17 highlighting aggregation phenomena.

The single cryoprotectants, their mixture and resuspending conditions were submitted to a Mixture design study using Statgraph software.

**Figure 4:**
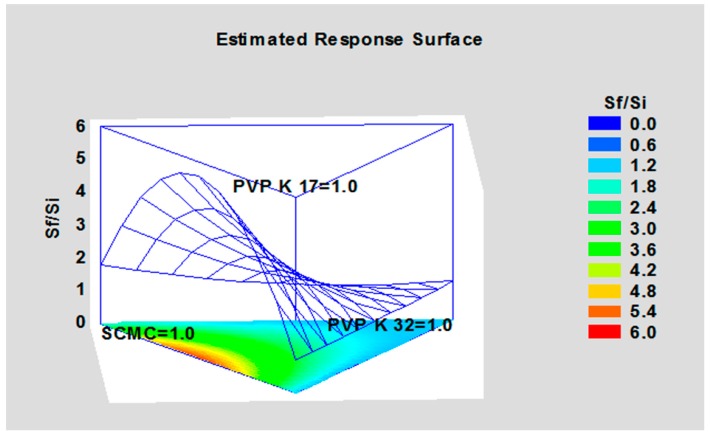
Response Surface of the Mixture design using the quadratic model.

Results are plotted in a simplex centroid, mixture design by statgraphics software (Figure 4). PVP K17, PVP K32 and SCM correspond to vertex. Binary mixture and ternary mixture combining the three cryprotectans must give a total amount that correspond two times the weight of the nanoparticles. The most appropriate model for this mixture design is a special cubic design because the R-squared is 99.88%, while linear and quadratic designs show a R-squared of 20.97% and 97.66%, respectively. Response surface plot shows that SCM exhibits higher S_f_/S_i_ with respect to PVP K17 and PVP K32. 

Nps characterization all along the study took into account also zeta potential. As known from the literature, zeta potential, is an important indicator of colloid suspension stability, even if not the only one [18]. Generally colloids are stabilized by high surface repulsive forces corresponding to zeta potential values of ±30 mV. The gentamicin sulfate loaded Nps have always approximately neutral zeta potential, in the range +0.5–3.58 mV, corresponding to highly unstable suspensions. The datum justifies freeze drying step, in order to stabilize nanoparticles and permit their storage. Moreover, it should be highlighted that the gentamicin loaded Nps zeta potential is slightly positive whenever gentamicin content increases, probably because of positively charged drug molecules on Nps surface. On the contrary, gentamicin sulfate loaded Nps resuspended after freeze drying have always slightly negative zeta potential, due to cryoprotectant interaction. Indeed the zeta potential values account for nanoparticle structure consistency. The values obtained are considered suitable since it has been found in the literature that neutral zeta potential positively affects antimicrobial activity [19]. 

**Figure 5:**
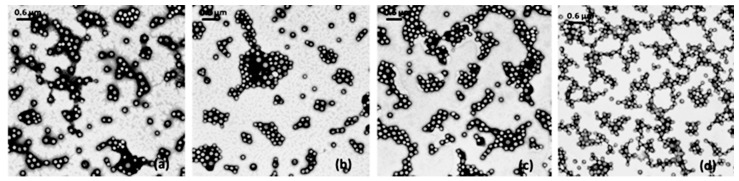
TEM micrograph showing the morphology of optimized gentamicin sulfate loaded nanoparticles batch #25: after centrifugation (**a**); freeze-dried without cryoprotectants (**b**); freeze-dried with PVP K17 (**c**); freeze-dried with a binary mixture of PVP K17/PVP K 32 (**d**).

Nanoparticles suspension (Batch #25) was analyzed by TEM before and after freeze-drying with and without cryoprotectants (Figure 5). Gentamicin sulfate loaded nanoparticles before freeze-drying were spherical in shape with average size of about 300 nm, confirming the data from dynamic light scattering. Nanoparticles freeze-dried without cryoprotectans addition show important aggregation phenomena. No variations of particle shape and size were highlighted for nanoparticles freeze-dried in presence of PVP K17 and mixture of PVP K 17 and PVP K 32 (Figure 5c,d). Nevertheless, sample freeze-dried with the binary mixture displays more inter-particle bridges linking nanoparticles.

**Figure 6:**
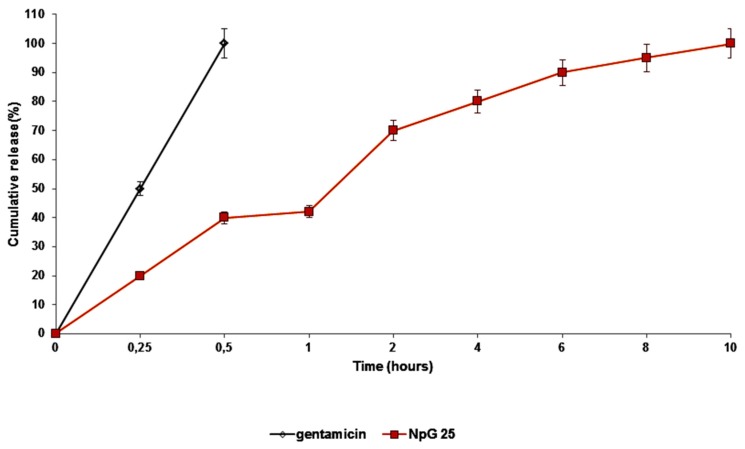
In vitro release profile of gentamicin sulfate from Batch #25 freeze-dried formulation, in PBS pH 7.4 at 37 °C, in sink condition. Gentamicin sulfate has been used as control.

The release of gentamicin sulfate from nanoparticles (batch #25) was evaluated in PBS pH 7.4 in order to mimic physiologic conditions. Gentamicin sulfate loaded Nps show a biphasic release profile with nearly 40% of gentamicin released after 1 h and 70% after 2 h. The complete release was reached in 10 h (Figure 6).

Following plots were made for kinetic study: cumulative% drug release vs. time (zero order kinetic model); log cumulative% drug remaining vs. time (first order kinetic model); cumulative% square root drug release vs. time (Higuchi model) and log cumulative% drug release vs. log time (Korsmeyer-Peppas model).

The results of kinetic study are reported in Table 9 where R^2^ is correlation value, *n*, is release exponent. On the basis of the best fit with the highest correlation (R^2^) value, gentamicin sulfate loaded nanoparticles resulted to follow Higuchi model with release exponent value slope 0.5352. The *n* value indicates that the release mechanism is Fickian diffusion [20].

### 3.4. Antimicrobial Activity

**Figure 7:**
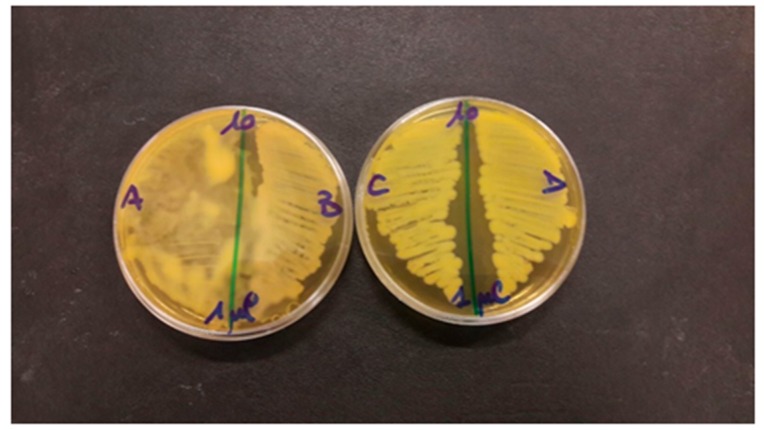
Bacterial growth upon coincubation with plabebo Nps of: (**A**) *Escherichia coli* ATCC 25922 incubated without Nps; (**B**) *Escherichia coli* ATCC 25922 incubated with placebo Nps; (**C**) *Pseudomonas aeruginosa* incubated without Nps; (**D**) *Pseudomonas aeruginosa* incubated with placebo Nps.

The bacterial survival test showed complete lack of any antibacterial activity when 20, 40, 70 and 80 µg/mL of placebo nanoparticles were added to a MH broth final volume of 1 mL. A visible turbidity and a 10^8/9^ CFU/ml bacterial grow on MH agar plates (agar plates 10 cm diameter) were always observed, as shown in Figure 7 for control *Escherichia coli* ATCC 25922 without Nps (A) vs. *Escherichia coli* ATCC 25922 incubated with placebo Nps (B), and *Pseudomonas aeruginosa* without Nps (C) vs. *Pseudomonas aeruginosa* incubated with placebo Nps (D). 

Minimum inhibitory concentration (MIC) and minimum bactericidal concentration (MBC) determination results are shown in Table 10. Susceptibility results were interpreted according to the EUCAST 2015 clinical guidelines and reported in brackets in Table 10, according to the three EUCAST categories susceptible (S), intermediate (I) resistant (R). EUCAST categories refer to clinical breakpoints for everyday use in clinical laboratories to advise on patient therapy. Therefore they give important information when clinical isolates are tested [15].

The MIC and MBC values of gentamicin sulfate loaded nanoparticles resulted to be generally equal to, or one dilution higher, than the ones obtained using free gentamicin. Standard *Escherichia coli* ATCC 25922 behaved similarly to clinical isolates. Gentamicin sulfate and gentamicin sulfate loaded Nps gave the same MIC results only towards *Staphylococcus aureus* 728. Gentamicin sulfate and gentamicin sulfate loaded Nps achieved the same MBC results towards *Escherichia coli* and *Proteus mirabilis*. Gentamicin sulfate loaded Nps showed lower MBC values (8 μg/mL) towards *Staphylococcus aureus* 728 with respect to free gentamicin sulfate (16 μg/mL). Considering clinical isolates variability it can be stated that no decrease in gentamicin sulfate MICs and/or MBCs values was highlighted testing gentamicin sulfate loaded nanoparticles. However, it has to be taken in account that in vitro presence of MH broth medium could negatively affect the interaction between bacterial cells and nanoparticles. 

## 4. Conclusions

On the basis of the present investigation it is possible to conclude that the preparation of gentamicin sulfate loaded nanoparticles by s/o/w technique is governed by several process variables, such as polymer concentration and composition, stirring rate, S/nS ratio, PVA concentration and addition of alcohols into PVA external aqueous solution. The results obtained in the systemic study performed on all these variables justify the following conclusions.

Using s/o/w technique the most important factors governing nanoparticle size resulted to be polymer composition, polymer concentration, stirring rate, S/nS ratio and PVA *w*/*v*%. 

Factors mostly affecting drug content resulted to be polymer composition and MetOH addition into external aqueous phase. DC of about 10.5 *w*/*w*% was achieved mixing PLGA-PEG polymer with PLGA-H, using a sufficient amount of surfactant (PVA) and reducing the dielectric constant of external aqueous phase by MeOH addition. These parameters are strictly related to the drug molecules characteristics. In case of gentamicin sulfate, its high water solubility and low molecular weight are issues to be overcome in order to achieve suitable Nps drug payloads.

Gentamicin release from the Nps was biphasic with about 40% of drug released in the first hour. The whole gentamicin release from Nps was prolonged 20 times with respect to free gentamcin dissolution rate.

Stabilization of gentamicin sulfate Nps freeze dried formulation involves addition of cryoprotectants. A mixture of PVP K17 and PVP K32 resulted to be the best cyoprotectant blend.

On the basis of the optimized process variables, gentamicin sulfate loaded nanoparticles were successfully synthesized with a good reproducibility and yield process. 

Gentamicin sulfate loaded nanoparticles maintain the drug antimicrobial activity at the same levels of free gentamicin as long as MIC and MBC values are concerned. The result is preliminary to a study on effect of gentamicin sulfate loaded nanoparticles on microbial biofilm. 

## Figures and Tables

**Table 1:** Factors and factor level studied in the screening experimental design (2^3^ = 8 batches).

Batch #	Polymer Conc. (mg/mL)	S/nS Ratio (*v*/*v*)	Stirring Rate (rpm)
1	12.5 (−1)	0.2 (−1)	350 (−1)
2	12.5 (−1)	0.5 (+1)	350 (−1)
3	12.5 (−1)	0.2 (−1)	700 (+1)
4	12.5 (−1)	0.5 (+1)	700 (+1)
5	25 (+1)	0.2 (−1)	350 (−1)
6	25 (+1)	0.5 (+1)	350 (−1)
7	25 (+1)	0.2 (−1)	700 (+1)
8	25 (+1)	0.5 (+1)	700 (+1)

**Table 2:** Runs parameters for the second full factorial, screening experimental design (2^3^ = 8 batches).

Batches #	Polymer Composition (PLGA-PEG/PLGA-H)	PVA (*w*/*v*%)	MetOH (*v*/*v*%)
9	70/30 (−1)	0.25 (−1)	30 (−1)
10	70/30 (−1)	0.5 (+1)	30 (−1)
11	70/30 (−1)	0.25 (−1)	60 (+1)
12	70/30 (−1)	0.5 (+1)	60 (+1)
13	30/70 (+1)	0.25 (−1)	30 (−1)
14	30/70 (+1)	0.5 (+1)	30 (−1)
15	30/70 (+1)	0.25 (−1)	60 (+1)
16	30/70 (+1)	0.5 (+1)	60 (+1)

**Table 3:** Mixture design; runs parameters for the stability study on freeze-dried nanoparticle formulations.

Batch #	PVP K17 *w*/*w* *	PVP K32 *w*/*w* *	SCM *w*/*w* *
1	2	0	0
2	0	2	0
3	0	0	2
4	1	1	0
5	0	1	1
6	0.66	0.66	0.66
7	0.66	0.66	0.66
8	0.66	0.66	0.66

* *w*/*w* is cryoprotectans and nanoparticles weight ratio.

**Table 4:** Effect of PLGA-PEG concentration, S/nS ratio and stirring rate on size, size distribution (PDI) zeta potential (mV) and drug content (DC).

Batch #	PLGA-PEG (mg/mL)	S/nS Ratio	Stirring Rate (rpm)	Size (nm)	PDI	Zeta Potential (mV)	DC *w*/*w*%	EE%
1	12.5	0.2	350	299.4 ± 54.4	0.266 ± 0.47	−1.06 ± 0.56	5.4 ± 0.70	43.97
2	12.5	0.5	350	384.6 ± 58.7	0.301 ± 0.43	−0.37 ± 0.98	7.7 ± 0.32	62.70
3	12.5	0.2	700	210.7 ± 42.4	0.104 ± 0.99	−1.28 ± 0.67	6.8 ± 0.86	54.39
4	12.5	0.5	700	140.0 ± 54.6	0.130 ± 0.57	0.36 ± 0.84	7.9 ± 0.45	64.33
5	25	0.2	350	855.5 ± 46.7	0.271 ± 1.28	−0.96 ± 0.88	2.9 ± 0.67	44.00
6	25	0.5	350	507.8 ± 47.9	0.176 ± 2.71	−5.23 ± 0.43	4.1 ± 0.67	63.07
7	25	0.2	700	381.5 ± 57.9	1.230 ± 0.24	−2.36 ± 0.75	3.7 ± 1.78	56.92
8	25	0.5	700	919.3 ± 53.2	0.138 ± 0.57	−5.54 ± 0.59	4.2 ± 1.68	64.61

**Table 5:** Nps preparation process: Optimization of organic phase and aqueous phase composition.

Batches #	Organic Phase Composition			Aqueous Phase			Results				
	PLGA-PEG (%)	PLGA (%)	Solvent	PVA (*w*/*v*%)	Alcholos		DC (*w*/*w*%)	Size (nm)	PDI	Z Potenzial (mV)	Process Yield (%)
					EtOH (*v*/*v*%)	MetOH (*v*/*v*%)					
4	100	0	Acetone	1	-	-	4.38 ± 2.45	240.0 ± 54.6	0.130 ± 0.57	0.36 ± 0.84	45 ± 2.34
9	100	0	DMSO	1	-	-	-	>1000 ± 14.5	1.214 ± 3.56	−7.13 ± 0.3	-
10	0	100	Acetone	1	-	-	27.31 ± 4.3	286 ± 43.9	0.02 ± 0.65	−3.18 ± 2.4	43 ± 5.8
11	30	70	Acetone	1	-	-	5.6 ± 2.3	326 ± 10.1	0.6 ± 0.78	−2.7 ± 1.1	45 ± 6.9
12	50	50	Acetone	1	-	-	0.94 ± 0.5	236 ± 25.4	0,01 ± 0.64	0.28 ± 0.4	43 ± 5.3
13	70	30	Acetone	1	-	-	0.2 ± 0.4	410.9 ± 2.6	0.26 ± 0.53	0.3 ± 0.3	42 ± 6.9
14	30	70	Acetone	0.5	-	-	1.54 ± 0.7	787 ± 59.5	0.57 ± 0.45	−1.86 ± 0.7	36 ± 8.3
15	30	70	Acetone	0.25	-	-	2.3 ± 0.2	801.5 ± 49.3	0.61 ± 0.32	−9.7 ± 0.4	42 ± 3.7
16	30	70	Acetone	0.5	30	-	19 ± 1.4	973 ± 23.4	0.71 ± 0.21	−0.1 ± 0.9	57 ± 5.4
17	30	70	Acetone	0.25	30	-	7.10 ± 2.3	672 ± 33.2	0.24 ± 0.26	−1.2 ± 0.5	40 ± 6.4
18	30	70	Acetone	0.25	20	-	5.47 ± 2.2	647 ± 39.1	0.275 ± 0.34	−1.02 ± 0.4	39 ± 4.8
19	30	70	Acetone	0.25	40	-	6.54 ± 2.6	763 ± 14.7	0.09 ± 0.54	−1.23 ± 0,9	42 ± 4.3
20	30	70	Acetone	0.25	60	-	54 ± 1.4	1000 ± 6.8	0.63 ± 0.39	−0.73 ± 0.7	62 ± 5.7
21	30	70	Acetone	0.25	-	30	11.59 ± 1.5	310 ± 11.7	0.13 ± 0.43	1.3 ± 0.6	60 ± 8.5

**Table 6:** Runs parameters and responses for 2^3^ (three factors at two level) full factorial screening design.

Batch #	PLGA-PEG/PLGA Ratio	PVA *w*/*v*%	MEtOH *v*/*v*%	Size nm	PDI	Zeta Potential	DC *w*/*w*%
22	70:30	0.25	30	365.5 ± 7.9	0.231 ± 0.66	0.67 ± 0.5	8.87 ± 2.3
23	70:30	0.5	30	643.9 ± 5.3	0.560 ± 0.75	0.34 ± 0.2	3.87 ± 2.4
24	70:30	0.25	60	711.6 ± 6.6	0.331 ± 0.67	−0.46 ± 0.3	4.22 ± 1.6
25	70:30	0.5	60	650.1 ± 8.5	0.520 ± 0.45	0.54 ± 0.1	6.54 ± 1.4
26	70:30	0.25	30	310.0 ± 11.7	0.130 ± 0.43	1.30 ± 0.6	10.59 ± 0.5
27	70:30	0.5	30	551.1 ± 9.5	0.260 ± 0.54	−0.61 ± 0.3	5.56 ± 2.0
27	30:70	0.25	60	876.4 ± 10.4	0.390 ± 0.32	0.44 ± 0.7	5.78 ± 1.3
28	30:70	0.5	60	480.2 ± 6.8	0.450 ± 0.21	−072 ± 1.0	4.57 ± 0.7

The predictive reduced model for DC is given in the equation, showing a R^2^ squared of 87.12%: DC = 9.73137 + 1.00125 × Polymer composition − 5.5165 × PVA (*w*/*v*%) − 5.2335 × MetOH (*v*/*v%*)+ 6.0755 × PVA (*w*/*v*%) × MetOH (*v*/*v%*).

**Table 7:** Resuspendability after centrifuge at 16,400 rpm, 4 °C for 20 min for optimized gentamicin sulfate loaded nanoparticles (batche #21).

Resuspension Conditions	Curing Conditions		Results				
	Temp. (°C)	Time (h)	Size (nm)	PI	DC *w*/*w*%	Resuspendability ***	Time (min)
A *	4	4	353.2 ± 15.4	0.1 ± 0.64	10.31 ± 1.5	±	30 ± 2.3
B *	4	5	330.0 ± 13.7	0.1 ± 0.72	9.85 ± 1.5	+	20 ± 1.1
C **	4	5	284.5 ± 10.7	0.15 ± 0.68	10.20 ± 1.5	+	12 ± 0.5

* Batch was resuspended in 200 µL of sterile water and maintained under agitation (30,000 rpm). ** Batch was progressively suspended in sterile water (100 µL + 100 µL), after each addition, the formulation was maintained under agitation for 60 s (30,000 rpm). Then suspension was sonicated for 5 min and further agitated for 5 min. *** Keys: (+) suspended nanoparticles, (−) complete polymer precipitation (no nanoparticle formation) and (±) mixture of suspended nanoparticles and polymer precipitation.

**Table 8:** Runs parameters and results of Mixture Design study.

Freeze-Drying Formulation	Cryoprotectants (*w*/*w*) *			Results		
	PVP K17	PVP K32	SCM	S_f_/S_i_ **	PI	Zeta Potential (mV)
1	2	-	-	1.0	0.179	−1.25
2	-	2	-	1.19	0.116	−1.50
3	-	-	2	1.8	0.564	−3.28
4	1	1	-	1.08	0.501	−0.34
5	-	1	1	1.17	0.934	−0.3
6	1	-	1	5.55	0.684	−0.274
7	0.66	0.66	0.66	2.42	0.355	−1.24
8	0.66	0.66	0.66	2.56	0.342	−1.56
9	0.66	0.66	0.66	2.31	0.450	−1.10

* mg cryoprotectants/mg Nps. ** S_f_/S_i_ Nps particles size before (S_i_) and after (S_f_) freeze-dried. S_f_/S_i_ = 1 absence of aggregation phenomena. S_f_/S_i_ > 1 presence of aggregation phenomena.

**Table 9:** Results of in vitro release model fitting for optimized gentamicin sulfate loaded nanoparticles (Batch #21).

Models	*n*	Slope	R^2^
Zero order	0.1039	0.85671	45.81
First order	0.015	0.77978	3.8281
Higuchi	3.2864	0.93953	24.539
Korsmeyer-Peppas	0.5352	0.79909	1.6538

**Table 10:** MIC and MBC for free gentamicin and gentamicin-loaded nanoparticles.

Tested Strains	MIC and MBC Values (µg/mL)/SIR Categorization (EUCAST)			
	Gentamicin Sulfate MIC (μg/mL)	Gentamicin Sulfate MBC (μg/mL)	Gentamicin Sulfate-Loaded Nanoparticles MIC (μg/mL *)	Gentamicin Sulfate-Loaded Nanoparticles MBC (μg/mL *)
*Escherichia coli*	2 (S **)	4 (I ^)	4 (I ^)	4 (I ^)
*Pseudomonas aeruginosa*	1 (S **)	2 (S **)	4 (R ^^)	8 (R ^^)
*Proteus mirabilis*	4 (I ^)	8 (R ^^)	8 (R ^^)	8 (R ^^)
*Staphylococcus aureus* 695	1 (S **)	1 (S **)	2 (S **)	2 (S **)
*Staphylococcus aureus* 728	8 (R ^^)	16 (R ^^)	8 (R ^^)	8 (R ^^)
*Escherichia coli* ATCC 25922	0.5 (S *)	0.5 (S*)	2 (S *)	2 (S *)

* μg/mL is referred to the concentration of gentamicin sulfate loaded into nanoparticles. ** S = susceptible. In EUCAST tables, the S category corresponds to S ≤ 1 mg/L. ^ I = intermediate. In EUCAST tables, the I category is not listed. It is implied as the values between the S breakpoint and the R breakpoint. I > 1–8 mg/L. ^^ R = resistant. In EUCAST tables, the R category corresponds to R > 8 mg/L.

## Supplementary Materials

The following are available online at http://www.mdpi.com/2079-4991/8/1/37/s1, Figure S1: DoE analysis of a full factorial design: Pareto chart and Estimated Response Surface for DC. Figure S2: DoE analysis of a full factorial design: (a) size distribution; (b) particle size.
