# Supplementary material for: Gentamicin Sulfate PEG-PLGA/PLGA-H Nanoparticles: Screening Design and Antimicrobial Effect Evaluation toward Clinic Bacterial Isolates
Source: Nanomaterials (Basel). 2018 Jan 12;8(1):37. doi: 10.3390/nano8010037 (PMC5791124; doi:10.3390/nano8010037)
Supplement: Supplementary file 1 [file nanomaterials-08-00037-s001.pdf]

*Article*

# Gentamicin Sulfate PEG-PLGA/PLGA-H Nanoparticles: Screening Design and Antimicrobial Effect Evaluation toward Clinic Bacterial Isolates

**Rossella Dorati [1], Antonella DeTrizio [1], Melissa Spalla [2], Roberta Migliavacca [2], Laura Pagani [2], Silvia Pisani [1], Enrica Chiesa [1], Bice Conti [1,*], Tiziana Modena [1] and Ida Genta [1]**

[1] Department of Drug Sciences, University of Pavia, Pavia 27100, Italy; rossella.dorati@unipv.it (R.D.); antonella.detrizio01@universitadipavia.it (A.DT.); silvia.pisani01@universitadipavia.it (S.P.); enrica.chiesa01@gmail.com (E.C.); tiziana.modena@unipv.it (T.M.); Ida.genta@unipv.it (I.G.)

[2] Department of Clinical-Surgical, Diagnostic and Pediatric Sciences, Unit of Microbiology and Clinical Microbiology, University of Pavia, Pavia 27100, Italy; melissa.spalla@unipv.it (M.S.); roberta.migliavacca@unipv.it (R.M.); laura.pagani@unipv.it (L.P.)

* Correspondence: bice.conti@unipv.it; Tel.: +39-0382-987-378; Fax: +39-0382-422-975

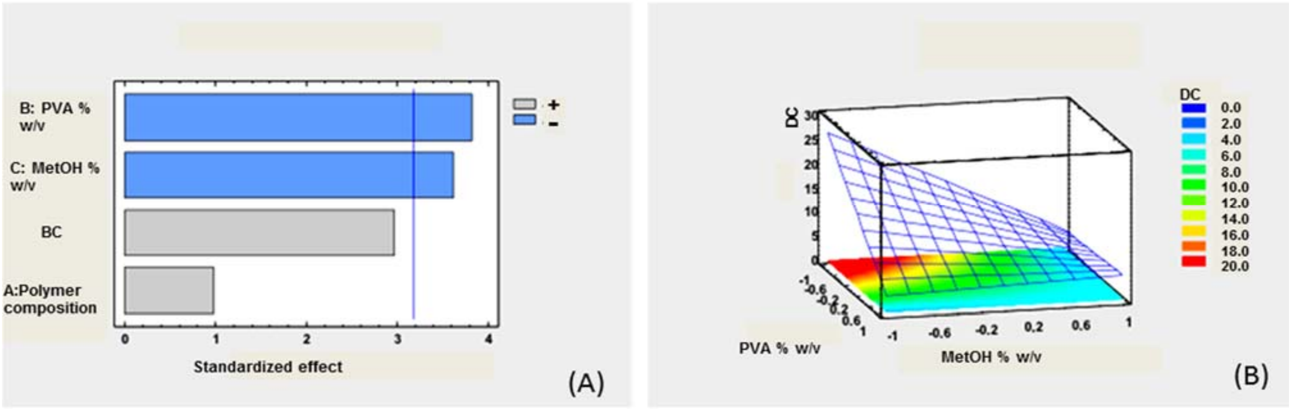


**Figure S1.** DoE analysis of a full factorial design: Pareto chart and Estimated Response Surface for DC: (A) standardized pareto chart for DC; (B) estimated response surface.

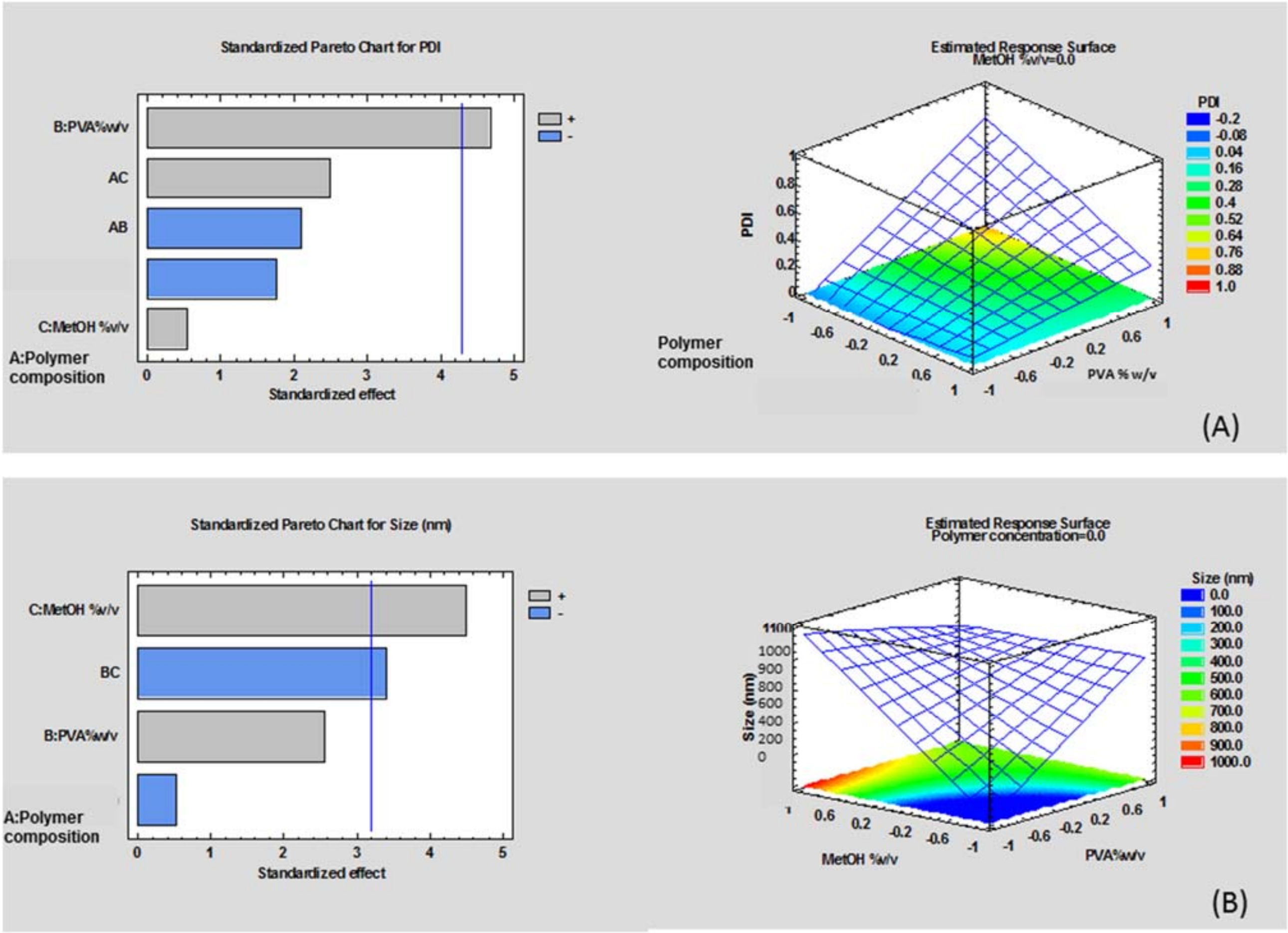


**Figure S2.** DoE analysis of a full factorial design: (**A**) standardized pareto chart for and estimanted response surface for PDI; (**B**) standardized pareto chart for and estimanted response surface for size (nm)
